# Polymorphism of the *XRCC1* Gene Is Associated with Susceptibility and Short-Term Recovery of Ischemic Stroke

**DOI:** 10.3390/ijerph13101016

**Published:** 2016-10-17

**Authors:** Wei He, Peng Huang, Dinghua Liu, Lingling Zhong, Rongbin Yu, Jianan Li

**Affiliations:** 1 Department of Epidemiology and Biostatistics, School of Public Health, Nanjing Medical University, Nanjing 211166, China; hewei861113@126.com (W.H.); hp19880310@163.com (P.H.); 2 Department of Rehabilitation Medicine, the First Affiliated Hospital of Nanjing Medical University, Nanjing 210029, China; 3 Department of Neurology, Medical College, The Affiliated Jiangyin People’s Hospital of Southeast University, Wuxi 214400, China; liudinghuajy@126.com; 4 Department of Neurology, Huai’an First People’s Hospital, Nanjing Medical University, Huai’an 223300, China; zll13299@163.com

**Keywords:** ischemic stroke, SNPs, XRCC1, base excision repair, prognosis

## Abstract

*Background:* Base excision repair (BER) is the primary DNA repair system with the ability to fix base lesions caused by oxidative damage. Genetic variants influencing the BER pathway may affect the susceptibility and the outcomes of ischemic stroke. Here, we examined how single nucleotide polymorphisms (SNPs) associated with BER impact susceptibility and short-term recovery of ischemic stroke. *Methods:* We selected 320 ischemic stroke patients and 303 controls. Then we genotyped SNPs of *NEIL1* rs4462560, *NEIL3* rs12645561 and *XRCC1* rs25487 in both groups. *Results:* Polymorphism in *XRCC1* rs25487 was significantly associated with reduced ischemic stroke (IS) risk (dominant model: OR = 0.53, 95% CI = 0.36–0.79, *p* = 0.002), a milder initial stroke (dominant model: OR = 0.57, 95% CI = 0.33–0.98, *p* = 0.043), and also a better short-term recovery (dominant model: OR = 0.57, 95% CI = 0.35–0.92, *p* = 0.022). No association was observed in the other two SNPs. *Conclusions:* Our study suggests that the genetic variant of *XRCC1* rs25487 may contribute to the etiology of ischemic stroke.

## 1. Introduction

Stroke is the second most common cause of death worldwide and the leading cause of death and adult disability in China [1,2]. Ischemic stroke (IS), a main type of stroke, is a heterogeneous disease, whose risk and clinical outcomes are influenced by multiple factors. Animal, clinical and epidemiological studies suggest genetic factors play an important role in stroke susceptibility and prognosis, in addition to conventional factors such as age, hypertension and diabetes mellitus [3,4,5].

Reactive oxygen species (ROS) are byproducts of the normal cell metabolism of oxygen, which may result in significant damage to DNA structures. The damaged DNA structures need to be restored by an integrated repair system. The role of ROS is complicated in ischemic stroke risks and outcomes. The high metabolism rates of the brain produce much more ROS. Chronic exposure to higher concentrations of ROS may damage the DNA of brain cells, which increase the risk of stroke. People with higher DNA repair activity may reduce such a risk. In addition, ischemia-reperfusion injury after acute ischemic stroke is also mediated by ROS [6]. Therefore, single nucleotide polymorphisms (SNPs) in genes encoding proteins involved in DNA repair can influence clinical outcomes following IS. Previous studies have indicated that *NEIL1*, *NEIL3* and *XRCC1* polymorphisms of the DNA repair pathway are involved in various pathologies, especially in cancers [7,8,9,10]. In this study, polymorphisms of the DNA repair genes *NEIL1* (rs4462560), *NEIL3* (rs12645561) and *XRCC1* (rs25487) were studied both as risk factors for the development of stroke and as modifiers of outcomes of ischemia.

## 2. Methods

### 2.1. Study Subjects

In this study, 320 patients (189 males and 131 females) with ischemic stroke and 303 controls were collected. Patients were recruited from the Department of Neurology, Jiangyin People’s Hospital and Huaian People’s Hospital, China. Controls were selected during the same period from Jiangyin People’s Hospital. According to the World Health Organization criteria, ischemic stroke was diagnosed [11]. All patients received computed tomography (CT) or magnetic resonance imaging (MRI) within 48 h after admission to the hospital. Blood vessels were evaluated using neck vascular ultrasound as well as CT angiography or magnetic resonance angiography. Conventional clinical hematology, biochemistry and immunology examinations were also conducted. Patients with atypical symptoms, including brain trauma, intracranial hemorrhage, post-seizure palsy, vascular malformations, metabolic disorders (except diabetes mellitus), infections, autoimmune diseases, blood diseases, cancers and severe chronic diseases (e.g., liver and kidney dysfunction) were excluded. According to Trial of ORG 10172 in Acute Stroke Treatment (TOAST) criteria, ischemic stroke can be divided into five subtypes: large-artery atherosclerotic stroke (LAA) and small-artery occlusive stroke (SAO), cardioembolic stroke (CE), stroke of other determined etiology (SOE), or stroke of undetermined etiology (SUE) [12]. Previous studies suggest that the strongest genetic influences would be detected in strokes attributed to large- or small-vessel disease [13,14]. Thus, this study mainly focuses on patients with those two subtypes. Both the cases and controls were free of atrial fibrillation, cardioembolism, and myocardial infarction. An informed consent which was approved by the Local Ethics Association and the Hospital Ethics Committee were signed by all patients (Project identification code: 2016-011).

### 2.2. Data Collection

A questionnaire was undertaken among both the case and control groups to assess risk factors. The information included demographic characteristics, medical history (hypertension, diabetes mellitus), history of alcoholism, daily cigarette smoking, obesity as well as parameters of hypercholesterolemia. Hypertension was defined as blood pressure (BP) ≥ 140/90 mmHg (average of three independent measures) or the use of antihypertensive drugs. Specially, in the diabetic subjects (68 IS patients and 38 controls) hypertension was defined as BP ≥ 130/85 mmHg (average of three independent measures) or the use of antihypertensive drugs. Diabetes mellitus was defined as fasting glucose level ≥ 7.0 mmol/L, a level ≥ 11.1 mmol/L 2 h after oral glucose challenge, or both, or receiving antidiabetic drugs. Subjects were considered as smokers if they smoked more than 10 cigarettes per day for five years, and as drinkers if they drank more than 50 mL alcoholic beverages per day for five years. Subjects with body mass index (BMI) ≥ 25 kg/m^2^ were considered as obese. The National Institutes of Health Stroke Scale (NIHSS) score and FIM instrument score were used to quantify stroke severity and functional independence of patients at the time of presentation and discharge. The FIM instrument is an 18-item scale, which measures independence involved in feeding, grooming, dressing, toileting, mobility, and cognition. It has proven to be responsive to small improvements in functional status after stroke [15]. Subjects are scored from 7 (totally independent) to 1 (totally dependent) on each item, with a score of 126 indicating total functional independence.

### 2.3. SNP Selection and Genotyping

Based on information in the NCBI SNP database and the International HapMap project data for the Han Chinese population and previous studies on DNA repair genes [16,17,18,19], the polymorphisms of rs25487 *(XRCC1*), rs4462560 (*NEIL1*) and rs12645561 (*NEIL3*) were selected. Fasting venous blood (10 mL, EDTA anticoagulant) was harvested from patients and controls. According to a standard protocol, total DNA from leukocytes was extracted using the salt fractionation method [20]. Single nucleotide polymorphism genotyping was performed with the TaqMan allelic discrimination assay on ABI PRISM 7900HT Sequence Detection system (Applied Biosystems, San Diego, CA, USA). Each genotyping assay contained one pair of primers and one pair of probes (sequences provided in Appendix A Table A1). Amplification was performed under the following conditions: 50 °C for 2 min, 95 °C for 10 min followed by 45 cycles of 95 °C for 15 s and 60 °C for 1 min. For quality control, five repeated samples and two blank controls were assigned into each 384-well format. The genotyping results were calculated by the allelic discrimination mode of the SDS 2.3 software package (Applied Biosystems, Foster City, CA, USA), and a 100% concordant was achieved.

### 2.4. Statistical Analyses

All statistical analyses were performed with Stata/SE (V.12.0 for Windows; StataCorp LP, College Station, TX, USA). Demographic data were compared using two-sample *t*-tests, chi-square (χ^2^) test. A goodness-of-fit χ^2^ test was used for Hardy-Weinberg equilibrium (HWE) among the controls. The association of genotypes with IS susceptibility, severity and short-term recovery and functional outcome were estimated by odds ratio (OR) and 95% CI using multivariate logistic regression analysis, with adjustment for age, sex, smoking, drinking, diabetes, hypertension, total cholesterol. All statistical tests were two sided and a *p* < 0.05 was considered as statistically significant. Bonferroni corrections were used for multiple comparisons.

## 3. Results

### 3.1. Clinical Characteristics of Participants

The detailed demographic and clinical characteristics of IS patients and controls are presented in Table 1. Compared to the control group, IS patients were significantly associated with a high proportion of smoking (*p* < 0.001), drinking (*p* < 0.001), suffering from hypertension (*p* < 0.001) and diabetes (*p* = 0.004). The patients also had higher serum triglycerides concentrations compared with controls. However, serum total cholesterol, serum HDL-C, serum LDL-C concentration or BMI failed to be identified as risk factors for IS in our study (Table 1). These observations confirm the role of smoking, drinking, hypertension and diabetes as risk factors for IS.

### 3.2. Association of SNP Genotypes with IS Susceptibility

As shown in Table 2, we detected all the three SNPs genotype frequencies in the 320 patients and 303 controls. None of the SNPs showed a significant deviation from HWE in the controls (*p* = 0.247, 0.530 and 0.231 for rs25487, rs12645561 and rs4462560, respectively). Only the SNP rs25487 showed a significantly different distribution between the patient and control group. There were no significant differences observed in the other two SNPs. After being adjusted for age, sex, smoking, drinking, diabetes, hypertension, and total cholesterol, logistic regression analysis indicated that *XRCC1* rs25487 variant genotypes had significantly reduced IS risk (dominant model: OR = 0.53, 95% CI = 0.36–0.79, *p* = 0.002). This decreased risk remained significant after Bonferroni correction.

### 3.3. Association of rs25487 SNP with IS Severity, Short-Term Recovery and Functional Outcomes

As the *XRCC1* rs25487 polymorphism was proved to be associated with IS susceptibility, we next investigated its association with IS severity and short-term recovery. The NIHSS score was dichotomized for logistic regression analysis as it was skewed and could not be transformed to normal distribution. Consistent with other studies [21,22], the cut-off for mild and severe IS was set between 6 and 7. Short-term recovery was defined as change in the NIHSS from presentation to discharge (ΔNIHSS). The short-term recovery score was also dichotomized for logistic regression analysis, as it was skewed. The cut-off was set at 0, where <0 means clinical improvement and ≥0 means no change or deterioration. As shown in Table 3, of the 320 IS patients, 228 individuals were classified as having mild IS and 92 as have severe IS. After being adjusted for age and sex, logistic regression analysis showed that patients harboring the rs25487 AG/AA genotype had a milder initial stroke (dominant model: OR = 0.57, 95% CI = 0.33–0.98, *p* = 0.043), and also showed a better short-term recovery (dominant model: OR = 0.57, 95% CI = 0.35–0.92, *p* = 0.022).

**Figure 1:**
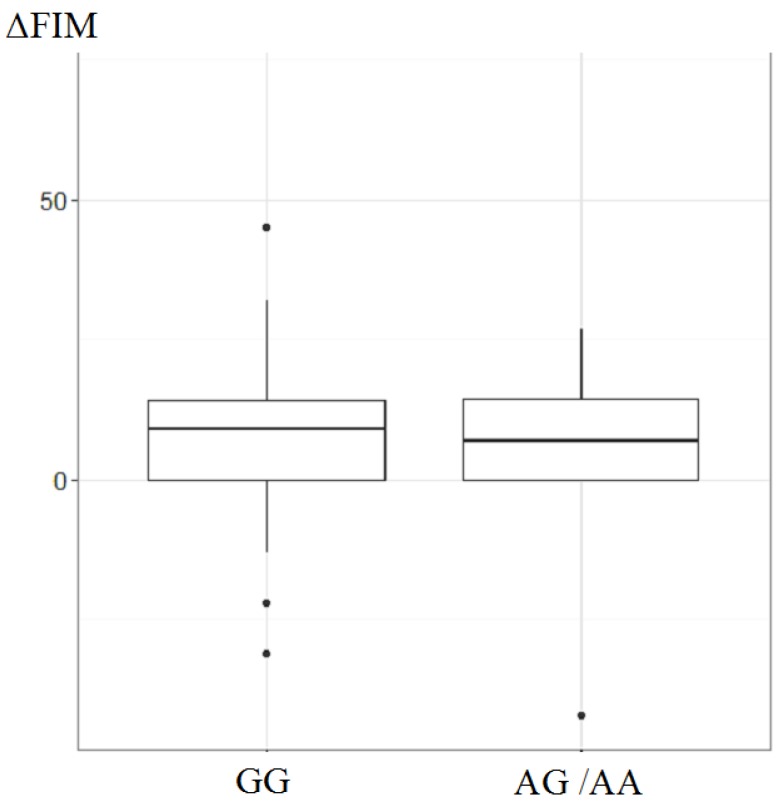
The relationship of the *XRCC1* rs25487 variant to the short-term functional outcome.

The relationship of the *XRCC1* rs25487 variant to the short-term functional outcome was next analyzed. Considering the few subjects with the AA genotype, we combined the AG and AA genotypes as one group. The short-term outcome was defined as the change in FIM scores from presentation to discharge (ΔFIM). As presented in Figure 1, the *t*-test analysis of ΔFIM scores showed no significant difference between the AG/AA group and the GG group (for AG/AA group, median: 7, IQR = 0–15; for GG group, median: 9, IQR = 0–14; *p* = 0.798).

## 4. Discussion

The capacity of cells for DNA repair may affect the susceptibility to stroke and influence the outcomes of stroke. Base excision repair (BER) is the primary DNA repair system with the ability to fix base lesions caused by oxidative damage, thus to maintain the integrity of the genome [16]. In this study, we investigated the associations between polymorphisms of three BER pathway genes and ischemic stroke.

There have been some studies concerning the relationship of the rs25487 polymorphism to IS susceptibility before. However, the results remain controversial. In a study involving 118 stroke cases, Somdat et al. reported that a polymorphism in the XRCC1*10 codon 399 (rs25487) was associated with a significantly reduced risk of stroke [18]. However, in another study involving 114 stroke patients, Shyu et al. found no significant association with IS susceptibility [19]. Our study suggested that the rs25487 mutation genotype was significantly associated with a decreased risk of ischemic stroke.

Accumulating evidence suggests that ROS-caused DNA damage following ischemia-reperfusion injury plays a critical role in neuronal cell death [16,23]. However, to the best of our knowledge, limited evidence is available to support the relationship of DNA repair genes to the functional outcome of IS patients, especially using detailed functional instruments such as FIM. Our study suggested that the rs25487 polymorphism was associated with acute IS severity and short-term recovery, although there was no association with short-term functional outcome. The inconsistency may result from the different emphasis points of NIHSS and FIM.

There are some limitations needing to be considered. The relatively small number of stroke cases may limit our ability to detect the association of SNPs with IS. Thus, we could not conclude that *NEIL1* or *NEIL3* polymorphisms have no important effect on IS. Moreover, the results were not replicated in independent subject panels. We should perform replication studies of the relation of these SNPs in independent and large subject panels. Our study only has a hint on the short-term recovery of patients; further studies are needed to address on the relationship of the SNPs to the long-term recovery and functional outcomes. Finally, our findings also need to be further independently verified in different ethnic populations.

## 5. Conclusions

In conclusion, this study showed that the genetic variant of *XRCC1* rs25487 was associated with a decreased risk of ischemic stroke. Furthermore, patients carrying the rs25487 variant genotype also had a milder acute severity and a better short-term recovery. Further studies are needed to elucidate the mechanism of the SNPs.

## Figures and Tables

**Table 1:** Demographic characteristics of patients and control subjects.

Characteristics	Cases (*n* = 320)	Control (*n* = 303)	*p* Value
Age, year (mean ± SD)	65.61 ± 11.12	67.11 ± 9.32	0.068
Sex (male) (%)	189 (59.1)	140 (46.2)	0.001
Smoking (%)	125 (39.1)	75 (24.8)	<0.001
Drinking (%)	151 (47.2)	64 (21.1)	<0.001
Diabetes (%)	68 (21.3)	38 (12.5)	0.004
Hypertension (%)	201 (62.8)	118 (38.9)	<0.001
BMI ≥ 25 kg/m^2^ (%)	114 (35.6)	101 (33.3)	0.554
Total cholesterol (mmol/L) (mean ± SD)	4.39 ± 1.12	5.19 ± 0.95	<0.001
Triglycerides (mmol/L) (mean + SD)	1.74 ± 1.45	1.56 ± 1.27	0.109
HDL-C (mmol/L) (mean ± SD)	1.24 ± 0.51	1.18 ± 0.26	0.075
LDL-C (mmol/L) (mean ± SD)	2.45 ± 0.95	2.65 ± 0.59	0.235

SD, standard deviation.

**Table 2:** Association of SNP genotypes with ischemic stroke (IS) susceptibility.

Genotype	Control (*n* = 303)	Cases (*n* = 320)	OR (95% CI)	*p* Value
rs25487				
GG	182 (60.1%)	212 (66.2%)	1.00	
AG	101 (33.3%)	102 (31.9%)	0.61 (0.41–0.92)	0.018
AA	20 (6.6%)	6 (1.9%)	0.16 (0.05–0.49)	0.002
Dominant			0.53 (0.36–0.79)	0.002
Additive			0.52 (0.37–0.74)	<0.001
rs12645561				
CC	162 (53.4%)	163 (50.9%)	1.00	
CT	122 (40.3%)	124 (38.8%)	0.97 (0.66–1.44)	0.898
TT	19 (6.3%)	33 (10.3%)	1.72 (0.85–3.49)	0.132
Dominant			1.07 (0.74–1.55)	0.707
Additive			1.15 (0.87–1.54)	0.328
rs4462560				
CC	88 (29.0%)	96 (30.0%)	1.00	
CG	160 (52.8%)	155 (48.4%)	0.76 (0.49–1.16)	0.196
GG	55 (18.2%)	69 (21.6%)	1.10 (0.64–1.89)	0.717
Dominant			0.84 (0.56–1.25)	0.395
Additive			1.01(0.78–1.32)	0.925

Logistic regression analyses adjusted for age, sex, smoking, drinking, diabetes, hypertension, total cholesterol.

**Table 3:** Association of *XRCC1* rs25487 polymorphism with IS severity and short-term recovery.

**(a) SNPs Associated with IS Severity**				
**Genotype**	**Mild (*n* = 228)**	**Severe (*n* = 92)**	**OR (95% CI)**	***p* Value**
rs25487				
GG	143 (62.7%)	69 (75.0%)	1.00	
AG	81 (35.5%)	21 (22.8%)	0.54 (0.31–0.95)	0.033
AA	4 (1.8%)	2 (2.2%)	1.12 (0.19–6.50)	0.897
Dominant			0.57 (0.33–0.98)	0.043
Additive			0.63 (0.38–1.05)	0.076
**(b) SNPs Associated with IS Short-Term Recovery**				
**Genotype**	**Improvement (*n* = 174)**	**No Change/Deterioration (*n* = 146)**	**OR (95% CI)**	***p* Value**
rs25487				
GG	105 (60.3%)	107 (73.3%)	1.00	
AG	65 (37.4%)	37 (25.3%)	0.57 (0.35–0.94)	0.026
AA	4 (2.3%)	2 (1.4%)	0.53 (0.09–3.00)	0.470
Dominant			0.57 (0.35–0.92)	0.022
Additive			0.60 (0.38–0.94)	0.026

Logistic regression analyses adjusted for age, sex.

## Appendix A

**Table A1:** Information of primers and probes for TaqMan allelic discrimination.

Polymorphism	Sequence (5’–3’)	
rs25487	Primer	F: AAGGAGTGGGTGCTGGACTGT
		R: CCAGCACAGGATAAGGAGCAG
	Probe	FAM-CCTCCCGGAGGTAA-MGB
		HEX-CCCTCCCAGAGGTA-MGB
rs12645561	Primer	F: CCTAGTTTCATGACTTGCTCATTACTG
		R: CAAAGGTCAGCTGTATTCAAAATCTATT
	Probe	FAM- AAGAAATAATACGCCTTGC-MGB
		HEX- ATAATACACCTTGCCAATT-MGB
rs4462560	Primer	F: ATGGCTTCCCAGGTATTTGGT
		R: GCTTCTCAACTCATGGTCTCTTCA
	Probe	FAM- ACCTCGAGGCACCA-MGB
		HEX- ACCTCGAGCCACCA-MGB
